# Characterization of the microenvironment of diabetic foot ulcers and potential drug identification based on scRNA-seq

**DOI:** 10.3389/fendo.2022.997880

**Published:** 2023-01-04

**Authors:** Yao Li, Shuai Ju, Xiaoyan Li, Wenqiang Li, Siyuan Zhou, Guili Wang, Yunmin Cai, Zhihui Dong

**Affiliations:** ^1^ Vascular and wound center, Jinshan Hospital, Fudan University, Shanghai, China; ^2^ Shanghai Medical College, Fudan University, Shanghai, China; ^3^ Department of vascular surgery, Zhongshan Hospital, Fudan University, Shanghai, China

**Keywords:** diabetes foot ulcers, microenvironment, dryness index, drug target, single cell sequence

## Abstract

**Background:**

Diabetes foot ulcers (DFUs) are a type of foot infection, ulcer, and/or deep tissue destruction caused by neuropathy and vascular disease in the distal extremities of diabetic patients. Its pathogenesis and its microenvironment are not entirely understood.

**Methods:**

Initially, the GSE165816 data set from the GEO database was utilized for single cell analysis to reveal the microenvironment and functional status of DFUs. The GSE199939 RNA-seq data set was utilized for external validation. On the basis of the logistic regression machine learning algorithm (OCLR), pseudo time series analysis, dryness index analysis, and drug target gene analysis were then performed. By constructing drug-gene and gene-gene networks, we can locate the most recent DFUs treatments. Finally, immunofluorescence technology was used to detect the cell-related markers of the DFUs microenvironment, and qPCR was used to detect the expression of drug targets in DFUs.

**Results:**

Firstly, we used the Cell Maker database to obtain information about human cells and related gene markers, and manually reviewed a total of 45 kinds of cells and maker information that may appear in the DFUs microenvironment, which were divided into 17 cell clusters after annotation. Subsequently, we counted the proportions of DM and DFUs in different types of cells, and the results showed that the proportions of macrophages, white blood cells, and monocytes were higher in patients with DFUs, while the proportions of pluripotent stem cells and stromal cells were higher in patients with DM. The Pseudo-time series analysis of cells in DFUs showed that the differentiation pathways of immune cells, mesenchymal cells and stem cells were similar in the three states, while the other cells were distributed in different stages. At the level of a single cell, the scores of both multipotential stem cells and hematopoietic stem cells were significantly lower in DFU healing and non-healing than in DM. Additionally, the highly expressed genes in DFU were chosen as drug targets. We identified seven potential target genes and discovered twenty drugs with high significance. Finally, the colocalization relationship between CD19, ITGAM, and HLA-DR expression in monocytes and macrophages of DFU skin tissue and healthy subjects was analyzed by laser confocal microscopy with the immunofluorescence triple labeling method. The results showed that the expressions of CD19, ITGAM, and HLA-DR in the skin of DFUs were significantly higher than those in the skin of healthy subjects, and the co-localization relationship was significant in DFUs.

**Conclusion:**

This study can serve as a resource for the treatment of DFUs.

## Introduction

Diabetes foot ulcers (DFUs) are one of the most severe diabetic complications (1). Its etiology involves numerous factors, including hyperglycemia-induced nerve damage, abnormal microvascular blood circulation, repeated infection, and difficult wound healing, among others (2). It is noteworthy that its occurrence plays a crucial role in diabetes-related vascular disease, neuropathy, and infection (3). According to research, about a quarter of DM patients will develop DFUs over the course of the disease. Every 20 seconds, a DFU-related amputation occurs somewhere in the world (4, 5). Even higher than colon cancer, breast cancer, and other malignant tumors, the five-year mortality rate is as high as 40 to 50%. DFUs are treated conservatively and rise early (6, 7). The drug is resistant to oral administration over the long term, and its effects are widespread. In cases of resting pain, surgical treatment is frequently necessary. Methods for reconstructing blood flow to the lower extremities include arterial bypass, stent implantation, and other surgical and interventional procedures (8). The use of spinal cord stimulation (SCS) to treat ischemic diseases was first reported in 1976 (9). It can help relax the blood vessels of the lower limbs, improve the microcirculation of the extremities, prevent gangrene and ulcers to some degree, promote their healing and repair, and lower the amputation rate (10). However, whether surgery or SCS treatment is used, it cannot completely rebuild blood vessels or treat only vascular diseases, and the rate of long-term recurrence is high (11). Because of this, it is important to look into new treatments and medicines for DFUs and figure out what role they play in the microenvironment of DFUs.

Currently, traditional intervention methods and DFU efficacy are unable to meet expectations (12). Stem cells can induce differentiation and secrete cytokines to form a capillary network in local ischemic tissues, establish collateral circulation, increase blood perfusion flow, and improve the ischemic condition of patients’ tissues in order to achieve a therapeutic effect (13, 14). Even as early as 2002, Yuyama et al. (15) was achieved using stem cell transplantation to treat ischemic diseases of the lower limb. His team’s research indicates that stem cells can differentiate into vascular endothelial cells and vascular smooth muscle cells (16). Moreover, they can secrete a large number of angiogenic factors, which expedite the delivery of stem cells to ischemic lower limbs. However, 85% of all DM amputees are caused by ulcers, and 15% of DM patients will develop lower limb ulcers during their lifetimes (17). DFUs are difficult to heal, and the disease’s progression is lengthy and complicated. Therefore, it is imperative to discover stem cell active agonists and therapeutic drugs for DFUs wounds (18).

This article retrieves the GSE165816 DFUs patient dataset from the geo database (19). GSE165816 is a DFU single cell sequencing data set with a large sample size; therefore, it was chosen for single cell sequencing analysis. Then, we selected the GSE199939 RNASEq dataset to validate our analysis results. We revealed the microenvironment of DFUs through exhaustive bioinformatics analysis and analyzed the cellular differences between DFUs and DM using pseudo-temporal analysis. In addition, we discussed the OCLR machine algorithm-based cell stemness index (20). Finally, we exported drugs through drug target genes in the drug network and found relevant drugs to treat DFUs. In conclusion, this research can serve as a new guide for the treatment of DFUs.

## Methods

### Acquisition and processing of data from a single cell

Data on single-cell RNA sequencing (GSE165816 dataset) was acquired from the group of Georgios Theocharidis. The sequencing of 94,325 single cells from 33 DFUs samples resulted in the retention of 94,325 single cells after data processing, including quality control, data filtering, and normalization. In this study, only processed transcripts from foot skin samples of 11 healthy subjects (including 8 diabetics), 9 DFU healing, and 5 DFU non-healing were used. Cells were sequenced using the Illumina HiSeq 4000 platform (Illumina, California, USA).

### Partial threshold settings

For downstream principal component analysis (PCA) and t-distributed stochastic neighbor embedding (t-SNE) analysis, Seurat (Version 4.0.2) was configured with partial threshold settings. Cells with fewer than 200 genes were eliminated from consideration. Gene expression was scaled after being normalized with the LogNormalize method. Following data normalization, 2000 highly variable genes (HVGs) were identified for each sample using the “VST” method. Subsequently, PCA was used to identify significant principal components (PCs), and the JackStraw and ScoreJackStraw functions were used to visualize the P-value distribution. The “IntegrateData” function was used to correct batches in order to avoid the batch effect of sample identity, which could disrupt subsequent analysis. In the end, 30 PCs were chosen for t-SNE analysis. With a resolution of 0.8, the FindClusters function was used to classify the cells into eight distinct clusters. To identify differentially expressed genes (DEGs) for each cluster, the FindAllMarkers function was used with logfc.threshold = 1 and p-value <= 0.01.

### The fundamentals of cellular annotation

Cellular annotation of DM and DFUs data We utilize the cellMaker database (http://xteam.xbio.top/CellMarker/ or http://bio-bigdata.hrbmu.edu.cn/CellMarker/) for cell and maker information. Cells that might be in the microenvironment of the DFUs were examined by hand and put together with cells of the same type. Using Fisher’s exact test and Jacquard’s coefficient, the characteristic genes and cytomaker genes of each cluster were used to annotate cells. The cluster was annotated as the cell type with the lowest P value (P <= 0.05) and the highest Jacquard coefficient. If there is no cell type with a p-value P <= 0.05, it is labeled as unknown.

### Cell locus investigation of DM and DFUs data

The package Monocle (Version 3.0) was used to figure out the path of the cells, get count data from the samples, and figure out the negbinomial. The size method was utilized to normalize the data. The genes were constructed using the differential genes in each cluster as the locus, with QVAL < 0.01 serving as the selection criterion for differential genes. The dimension of the data was then reduced using the reverse graph embedding (DDRTree) algorithm, and the quasi-timing analysis was performed using the orderCells function. Using pseudotime and cell type, the plot cell trajectory function allows for the visualization of cell trajectory inference.

### Analysis of gene ontology and Kyoto encyclopedia of genes and genomes enrichment

Gene Ontology(GO)and Kyoto Encyclopedia of Genes and Genomes(KEGG) are gene function and structure enrichment databases, respectively. In sequencing the genome or transcriptome, it is common to find different genes in various individuals or populations. The analysis of differential genes may reveal the causes of individual or group differences. Differential genes can be enriched in biological processes, cell distribution, structure, and function *via* GO enrichment. During enrichment, each enrichment term has a GO ID number. The unique GO ID number can be obtained through the enrichment of differential genes, followed by the enrichment of those genes into various pathways. KEGG is mostly about signaling pathways, and its database can add different genes to different pathways.For GO\KEGG enrichment analysis of DFUs and healthy subject-specific genes, clusterProfiler packages were utilized to identify significantly enriched pathways with a p <= 0.05 cutoff, and the top 20 pathways were chosen for display.

### Bulk gene expression data acquisition and processing

The GEO database’s TPM data for GSE199939 was extracted using the GEOquery package. Using the limma package, DEGs were determined. Significantly dysregulated genes were determined to have a P value < 0.05 and an absolute log2(FC) > 1.

### Dry analysis of individual cells and bulk samples

All expression data of a specific cell type were first extracted for dry evaluation at the level of a single cell, and then all expression values of the same cell in the same patient were averaged to form the gene expression data of the cell in the patient.The OCLR algorithm is a logistic regression machine learning algorithm (OCLR, One Class Linear Regression) published in Cell in 2018 that measures the level of moisture in tumor samples. In this paper, the microenvironment and relative humidity of DFUs are evaluated. We characterized sample dryness at the level of gene expression using mRNAsi scores. Human stem cell datasets provided by the Progenitor Cell Biology Consortium (PCBC) (https://www.synapse.org) were used as training data. Following training, the dryness of hematopoietic stem cells and pluripotent stem cells in ulcer microenvironments of DFUs and DM patients was evaluated, as well as the dryness of bulk-level samples.

### Construction and investigation of the drug-gene network

The DGIdb database (https://www.dgidb.org/) was utilized to obtain drugs associated with potential drug target genes, as well as other genes associated with this drug. Using bulk data, the Spearman correlation coefficient between drug-related genes was calculated, and significant co-expressed gene pairs were identified using the criteria p <= 0.05 and COR > 0.70. Thus, drug-gene and gene-gene pairs were generated to construct the drug-gene network, which was visualized using cytoscape 3.8.2. For the drug-gene network, potential drug targets were used as seed nodes to evaluate the significance of drugs using the WGCNA algorithm. The chance of starting over is 0.1, and the iteration keeps going until the difference between this iteration’s results and the results of the previous iteration is 10-10. This is done to figure out how much each drug weighs.

### Clinical sample collection

From March 2021 to January 2022, 22 patients with DFUs admitted to our hospital were recruited as the DFU group, including 12 males and 10 females, with a mean age of 56.12 ± 12.93 years and a mean duration of diabetes of 3–24 years. All patients lacked complications such as malignant tumors, heart, liver, or kidney dysfunction, or a history of cerebral hemorrhage, cerebral infarction, or acute myocardial infarction (AMI). In addition, 12 healthy individuals who underwent physical examination in our hospital during the same time period were recruited as the healthy control (HC) group. This group consisted of nine males and three females, with an average age of 58.23 ± 15.48 years. In terms of gender ratio and age, there was no significant difference among the three groups (all P > 0.05). In the morning, 10 ml of fasting blood was drawn from each subject’s cubital vein into a heparin anticoagulant tube, centrifuged for 20 minutes at 3500 rpm/min, and the serum was separated and stored at -80°C. Six cases of foot skin tissue from diabetic foot ulcers and six cases of healthy people were chosen and fixed with formalin. Immunofluorescence detection was then used to find the disease.

### Immunofluorescence method

The paraffin sections were put into water and placed in a repair box filled with citric acid (PH6.0) antigen repair solution in a microwave oven for antigen repair. After the sections were slightly dried, a tissue chemical pen was used to draw a circle around the tissue, and the primary antibody and secondary antibody were added and incubated for 50 minutes at room temperature in the dark. The slides were placed in PBS (PH7.4) and washed on a decolorization shaker for 3 times, 5 minutes each time. The auto fluorescence quencher was added to the ring for 5 minutes and rinsed with running water for 20 minutes. DAPI counter stained nuclei were slightly dried and then sealed with anti-fluorescence quenched tablets. Then the sections were observed under a fluorescence microscope and images were collected.

### qRT-PCR

Using the TRIzol reagent, total RNAs from the plasma were extracted and reverse-transcribed into cDNA. The PCR reaction was comprised of 40 cycles of denaturation at 95°C for 10 minutes, annealing at 60°C for 1 minute, and extension at 95°C for 15 seconds. As the internal standard, Glyceraldehyde 3-phosphate dehydrogenase (GAPDH) was utilized. The levels of related factors’ expression were determined using a PCR reaction with 40 cycles of denaturation at 94°C for 15 minutes, annealing at 55°C for 30 seconds, and extension at 70°C for 1 minute. GAPDH was used as an internal reference. Each experiment was conducted a minimum of three times. Using the 2-Ct (cycle threshold (CT)) formula, the relative concentrations of ANPEP, BID, CYBA, CYBB, FCER1G, ITGA1, and PLAUR were calculated.

## Results

**Figure 1:**
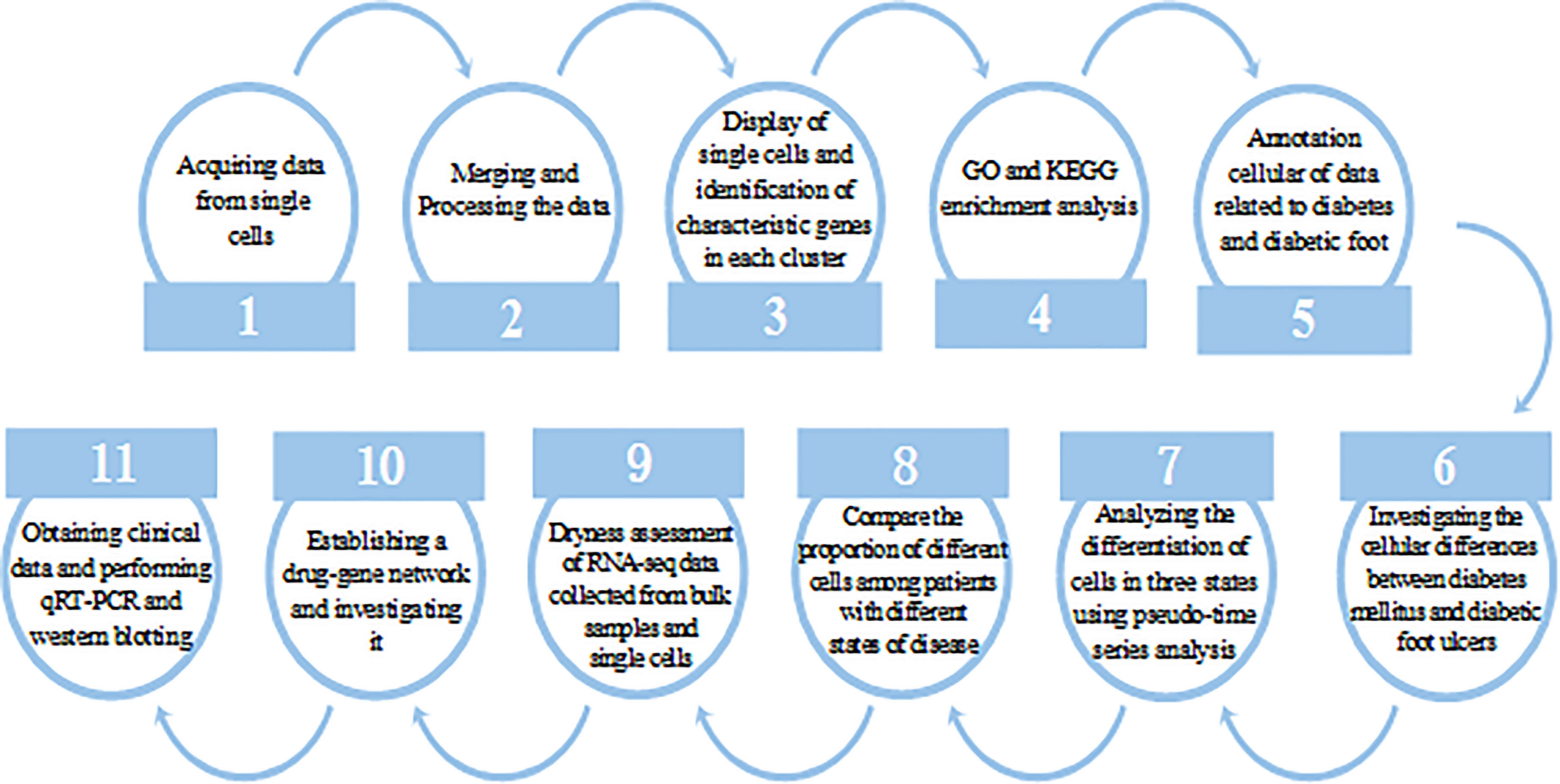
Research work flow chart.

Our workflow diagram is depicted in **Figure 1**.

### Single cell sequencing data analysis

**Figure 2:**
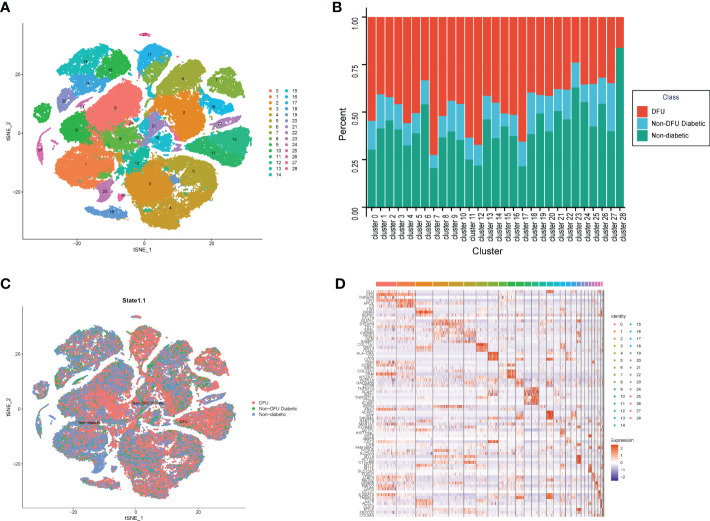
The single cell atlas of DFU, Non-DFU diabetic and Non-diabetic. **(A)** The clusters of single cell atlas using the t-SNE for visualization. **(B)** The spinogram of the percentage of different cell subtypes (c0-c28) in different groups (DFU, Non-DFU and Non diabetic). **(C)** The cell clusters split by different groups (DFU, Non-DFU and Non diabetic). **(D)** The gene expression heatmap of top mark genes of each cell clusters.

To integrate different samples, we first analyzed the single-cell sequencing dataset GSE165816 from DUF. Using the k-nearest neighbor (KNN) clustering algorithm, 94,325 cells were grouped into 29 clusters (**Figure 2A**). Single cells were displayed according to sample selection and sample state, as shown in **Figure 2C**. The results demonstrated that cells from various sample sources were essentially mixed together, and the integration effect between 33 samples was good with no discernible batch effect, which was utilized for further analysis. When comparing the distribution of single cells in patients with various states, it was discovered that patients with normal and DFUs were essentially separated, whereas patients with DM were dispersed between them. On the one hand, the microenvironment of DFU patients is very different from that of normal patients. On the other hand, this suggests that the microenvironment of DM patients changes gradually from normal to DFUs.

We also determined the proportion of patients from various states in each cluster. Consistent with the tSNE dimensionality reduction diagram, the results were The proportion of diabetic patients was average and not prominent in all clusters, whereas the proportions of normal and DFU were quite high in some clusters (**Figure 2B**, **Table S1**). Cluster7, Cluster11, Cluster12, and Cluster17 are DFU-specific clusters, accounting for 33%, 46%, 63%, and 51%, respectively. Clusters 23 and 28 are distinct clusters of healthy subjects, accounting for 46% and 40% of the sample, respectively. Then, based on the different clusters of different samples, the 2510 genes that were unique to each cluster were found (**Figure 2D**, **Table S2**).

### Analysis of skin function improvement in normal individuals and DFU

**Figure 3:**
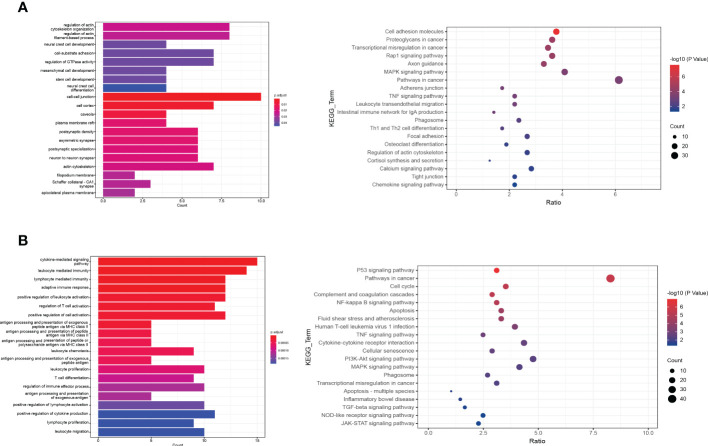
The functional enrichment analysis of DFU genes. **(A, B)** The bar plot and dot plot show the result of GO (Gene ontology) enrichment analysis.

To investigate the differences between normal and DFU skin, we identified additional characteristic genes in normal and diabetic feet using p <= 0.01 and ABS (logFC) > 1 as the threshold and then intersected them with characteristic genes corresponding to specific clusters. In the skin of normal people’s feet, 64 characteristic genes and 77 DFU characteristic genes were identified. A GO functional enrichment analysis was performed (**Figures 3A, B**).

### Skin microenvironment evolution in diabetic and non-diabetic feet

**Figure 4:**
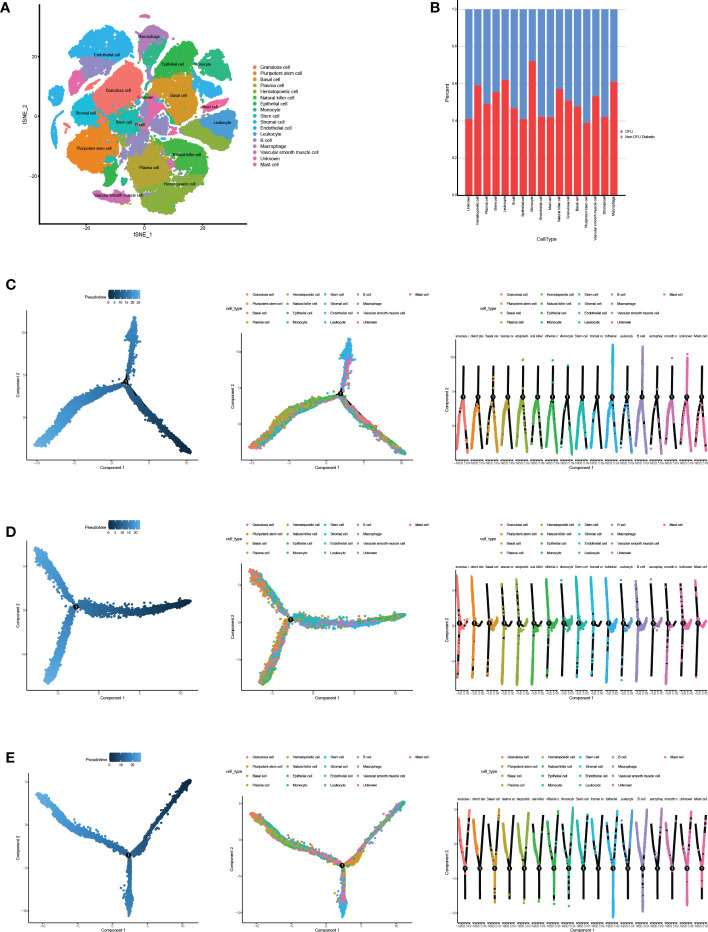
Cell annotation and pseudotime analysis. **(A)** Classifying the different cells on the t-SNE plots. **(B)** The heatmap shows the annotated cell abundance within different disease samples (DFUs and Non DFUs diabetic).**(C-E)** Pseudo-time analysis of DFUs healing, non-healing samples and togethers. Pseudotime, cell development orders.

The information about human cells and related gene markers, as well as the information about 45 types of cells and makers that may appear in the DFU microenvironment, was retrieved manually. The expression of established cell-specific marker genes assisted in classifying these 45 clusters of cells as 17 distinct cell types. We identified the most common cell types found in human skin. A variety of cells are included in this study, including granulosa cells, induced pluripotent stem cells, basic cells, plasma cells, hematopoietic cells, natural killer cells, epithelial cells, monocytes, stem cells, strategic cells, endothelial cells, white blood cells, B cells, large phase cells, smooth muscle cells, unknown cells, and mast cells (**Figure 4A**, **Table S3**).

To further investigate the distinction between diabetic ulcers and DFUs, we determined the proportion of diabetes and DFUs in various cell types (**Figure 4B**). According to the results, macrophages, leukocytes, and monocytes made up 65%, 67%, and 68%, respectively, of DFUs. In diabetic patients, the percentages of pluripotent stem cells and endothelial cells were 18% and 17%, respectively. We hypothesized that DFUs would be associated with increased ulcer microenvironmental inflammation and decreased dryness. We performed a pseudo-temporal analysis of DFUs’ healing and non-healing samples in order to determine the causes of this phenomenon. The results showed (**Figures 4C–E**) that immune cells, mesenchymal cells, and stem cells had similar differentiation pathways in the three ulcer microenvironment states, while other cells were distributed at different stages.Therefore, we hypothesized that the differences in the skin microenvironment of the three groups of feet were not due to differences in the timing of cell appearance but rather to differences in the number and activity of specific cells.

### Specific cell abundance in different samples

**Figure 5:**
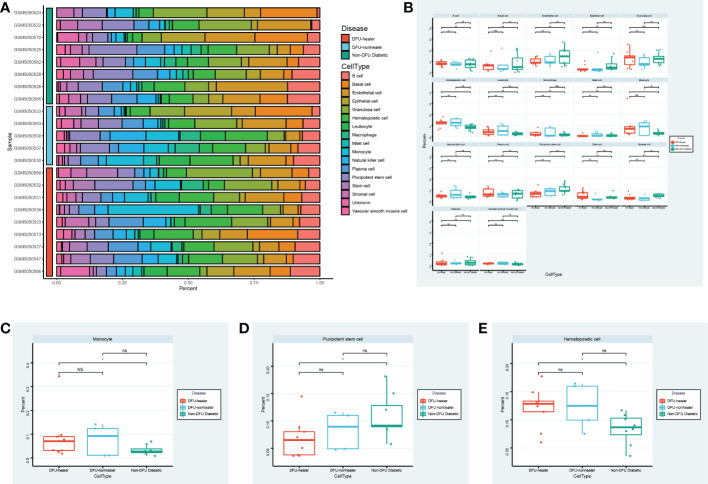
Specific cell abundance in different samples. **(A)** The heatmap shows the annotated cell abundance within different disease samples. **(B)** The box plots compare the differences in the percentage of each cell type between the different groups. Specially, **(C, D, E)** show respectively the differences in the percentage of Monocyte cells, Pluripotent stem cells, Hematopoietic cells between the different groups. * p-value < 0.1 ** p-value < 0.05 *** p-value < 0.001 ns, no significance.

Utilizing single-cell sequencing analysis, we analyzed the proportion of specific cells in various sample types (**Figure 5A**). While there were significantly fewer pluripotent stem cells in DFU than in diabetic patients, there were significantly more macrophages in DFU than in diabetic patients (**Figures 5B–E**). This suggests that dryness and inflammation are two crucial factors in the development of diabetic feet. It is essential to remember that patients with diabetic foot union and non-union have higher hematopoietic stem cell concentrations than diabetic patients.

### Index analysis of diabetic podocyte stem cells

**Figure 6:**
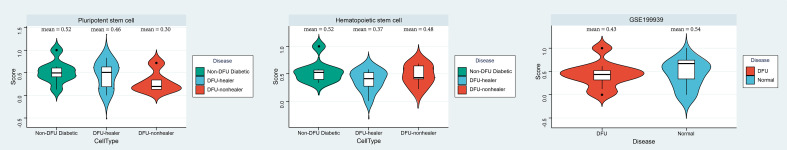
Index analysis of diabetic podocyte stem cells. Using the OCLR algorithm, the dricity of hematopoietic and pluripotent stem cells in DFU healing and non-healing as well as diabetic patients was assessed, and Violin diagram showing the differences between disease groups (left and middle). Furthermore, GSE199939 Validated the dricity between the DFU and Normal tissue (right). Score, dry scores.

According to the results of the aforementioned analysis, even though hematopoietic stem cells were prevalent in glycofoot, their activity may have been low. Using the OCLR algorithm, we evaluated the dricity of hematopoietic and pluripotent stem cells in DFU-healing and DFU-non-healing patients as well as diabetic patients (PMID: 29625051). As a measure of the gene’s overall expression in the DFU microenvironment, the average expression of a gene in a patient’s pluripotent stem cells or hematopoietic stem cells was used. The OCLR algorithm was then utilized to assess the hematopoietic and pluripotent stem cells of each patient (**Figure 6**, **Table S4**).Using the GEO database, we obtained a set of RNASEq data (GSE199939) from DFUs patients, and the patients in this set of data were graded for dryness to strengthen the conclusions. At the single-cell level, both pluripotent stem cells and hematopoietic stem cells in healing and non-healing DFUs had significantly lower dry indexes compared to those in diabetes (the mean dry index of pluripotent stem cells in healthy subjects was 0.52, the mean dry index of pluripotent stem cells in DFUs healing patients was 0.46, and the average dryness index of multipotent stem cells in DFUs healing patients was 0.31). The mean dryness index of hematopoietic stem cells was 0.54 in healthy subjects, 0.38 in DFU-healing patients, and 0.48 in DFU-non-healing patients. This was also confirmed at the bulk level, where the DFUs’ dry scores were lower than those of typical subjects.

### Screening potential compounds or activators that target dry characteristics using drug gene network analysis

**Figure 7:**
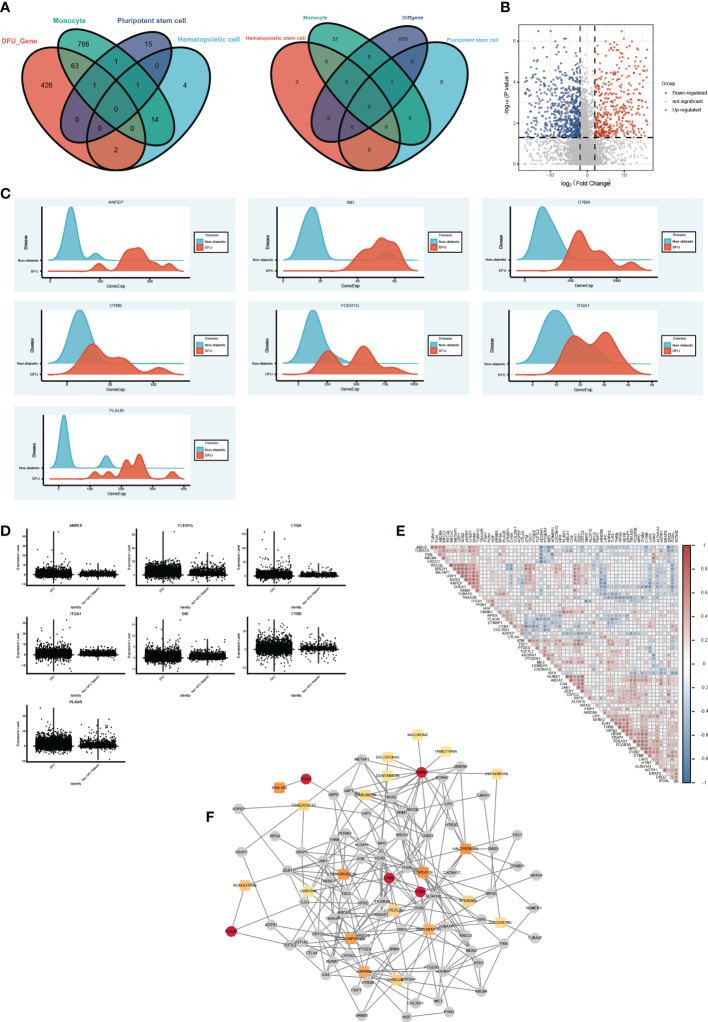
The drug gene network analysis. **(A)** The Venn diagram shows the intersections of DFU genes (left) and DEGs (right) between Monocyte cells, Pluripotent stem cells and Hematopoietic cells mark genes. **(B)** The Volcano plot shows the drug target differential expression genes. Blue, down- regulated, Red, up-regulated. **(C)** The ridge plot of drug genes between DFU and Non diabetic tissues. **(D)** The boxplot diagram of drug genes between DFU and Non diabetic tissues. **(E)** The Spearman correlation coefficient between medications and their intended targets. **(F)** Drug genes and drug gene networks using the DGIdb database.

Based on the aforementioned analysis, we discovered that monocytes, hematopoietic stem cells, and pluripotent stem cells are the primary distinct cell populations in the ulcer microenvironment of DM and DFU patients. In light of these three cell types, we were able to identify possible drug targets. Then, utilizing bulk data, we assessed the differences between the drug target genes that had been screened initially in DFUs and in healthy patients (**Figures 7A, B**). We subsequently screened the significantly overexpressed genes in DFUs as potential drug targets (**Figures 7C, D**, **Table S5**). Finally, seven potential target genes were identified: ANPEP, BID, CYBA, CYBB, FCER1G, ITGA1, and PLAUR. We then searched the DGIdb database for drugs that interacted with these genes. Using the bulk-level data, we calculated the Spearman correlation coefficient between the target genes. We also obtained additional information about these medications and their intended targets (**Figure 7E**). Drug genes and drug gene networks were developed in order to find more potential drugs (**Figure 7F**). Among the drugs with the greatest potential for treating DFU were CYCLOSPORINE, SIMVASTATIN, CURCUMIN, LUTEOLIN, APIGENIN, and CHRYSIN.

### Drug target genes are related to the immune microenvironment of DFUs

**Figure 8:**
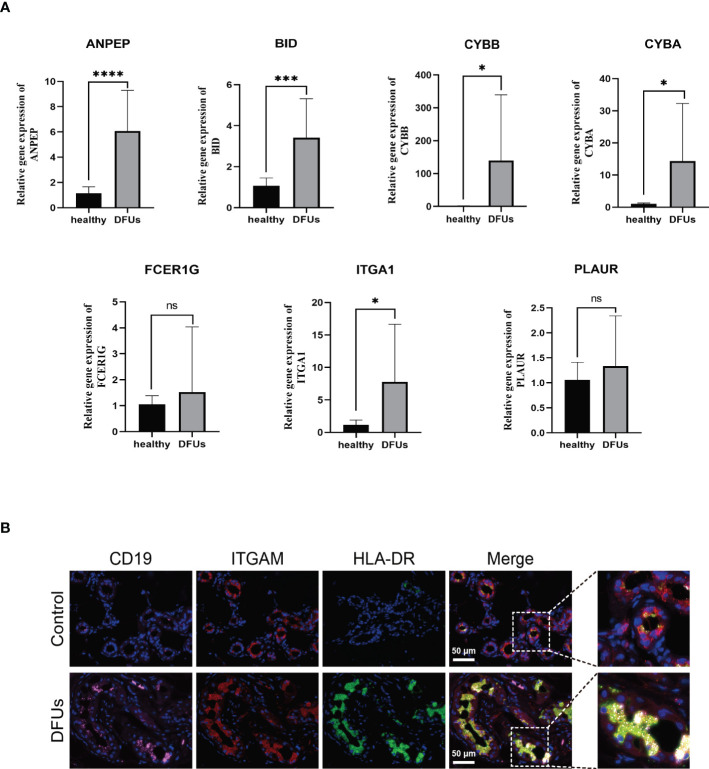
he relationship between drug target gene and DFU immune microenvironment was detected. **(A)** the expression levels of ANEP, BID, CYBA, CYBB, FCER1G, ITGA1 and PLAURmRNA in blood were detected for the first time by qRT-PCR. **(B)** the coexistence of CD19, ITGAM and human leukocyte antigen DR in DFU skin tissue and mononuclear macrophages was analyzed by laser confocal microscope and immunofluorescence triple labeling technique. ns, no significance.

Plasma tissues from 22 DFUs patients and 12 healthy subjects were clinically collected to further support the hypothesis that the genes screened above are related to the immune microenvironment of DFUs. ANPEP, BID, CYBA, CYBB, FCER1G, ITGA1, and PLAUR mRNA expression levels were first discovered in the blood by qRT-PCR. We noticed that the blood of DFUs patients had significantly higher expression levels of ANPEP, BID, CYBA, CYBB, FCER1G, ITGA1, and PLAUR (**Figure 8A**, **Table S6**). A number of microenvironment-related markers were also identified in skin tissues taken from six DFUS patients and healthy subjects using immunofluorescence. The colocalization relationship between CD19, ITGAM, and HLA-DR expression in DFU skin tissues and monocytes and macrophages of healthy subjects was analyzed by laser confocal microscopy and immunofluorescence triple labeling. The results showed that the expression of CD19, ITGAM, and HLA-DR in DFUs skin was significantly higher than that in healthy human skin, and the colocalization relationship in DFUs was significant. This was in line with the results of our single-cell sequencing, which showed that monocytes and macrophages were likely involved in the development and spread of DFUs (**Figure 8B**).

## Discussion

Diabetes patients frequently develop diabetic foot ulcers, also referred to as diabetic acrogangrene. Local tissue ischemia, neuropathy, and infection work in concert to almost completely predict its occurrence (21). Diabetes alters the osmotic pressure of the human body’s environment, and human blood vessels are vulnerable to pathological changes. This destroys the human microenvironment and increases the risk of ischemic neuritis or nutritional disorders as well as the development of diabetic foot symptoms (22). Infection is yet another significant factor in the development of diabetic feet (23). Diabetes patients often have impaired immune systems, and when combined with the foot’s position as one of the body’s most important organs and its propensity for trauma, infection can lead to further inflammation and, ultimately, the development of DFUs (24). One of the serious symptoms of diabetes that necessitates amputation is diabetic foot (25). The key to managing diabetes is to create a favorable microenvironment. In recent years, research and development have discovered a strong correlation between the development of diabetic feet and changes in the microenvironment (26). Changes in the local microenvironment cause some cellular elements and other factors in the local necrotic tissue to change, which has an impact on the development of ulcers and ultimately results in diabetic foot. We discovered that DF patients made up a significant portion of some clusters, and we discovered the characteristic genes for those clusters. The GO enrichment analysis of the distinctive genes in the DFUs cluster was also examined. These results could be a target for DFUs therapy, changing the pathological state of nearby foot wounds into a physiological state and creating a microenvironment that helps wounds heal.

Single-cell sequencing of tissues produced 94,325 cells’ worth of sequencing information. The age of diabetic and diabetic foot cells in various cell types was calculated. Leukocytes and monocytes were the most common types of cells in the DFUs cell population. In the diabetic cell population, pluripotent stem cells and stromal cells were the most common types of cells, showing that cells come from many different places. In the original paper about data mining, the author used 54 samples from 17 patients to conduct 21 cell clustering. In this study, smooth muscle cells, SMCs, fibroblasts, Fibro, vascular endothelial cells, VasEndo, T-lymphocytes, T-lympho, CD14+ monocytes, CD14-mono, differentiated keratinocytes, DiffKera, and basal keratinocytes, BasalK were analyzed. This is basically consistent with the study of DFUs microenvironment clustering cells in this paper. But the data we used was not completely consistent, and we only extracted 33 samples for analysis. In the process of cell annotation, annotation results are affected by annotation methods and maker sets. The dimensionality reduction clustering methods, annotation methods, and maker sets of our original text are all different, so the results may be different to some extent. But there is no gold standard for judging the accuracy of annotation, and the results of our cell annotation are based on the results of calculations and manual review, so it is reasonable to think that it has some accuracy.

In addition, we discovered that in the three ulcer microenvironments of diabetes mellitus, DFUs non-union stage, and healing stage, macrophages, leukocytes, and monocytes appeared earlier, stromal cells and pluripotent stem cells appeared later, and other cells were distributed at all stages. The majority of macrophages, according to the most recent research, are derived from blood monocytes, with the remainder coming from fetal liver monocytes and hematopoietic stem cells in the original hematopoietic tissue. From directional stem cells in bone marrow, monocytes gradually develop and mature into peripheral blood, enter the tissue through the vascular wall, and eventually develop into macrophages (27). The ability of cells to phagocytose is gradually improved during this process, and many soluble factors are also secreted (28). Under the stimulation of various pathogen-related molecular models, PAMP and DAMP, macrophages were polarized into M1-type macrophages (classical activation pathway macrophages) and M2-type macrophages (alternative activation pathway macrophages) (29, 30). A macrophage phenotype exception occurs in diabetic patients with high blood sugar levels, local microenvironment change, local ischemia, and hypoxia; the number and proportion of M1 macrophages increased, the secretion of inflammatory factors levels increased, but their stimulus following activation of inflammatory factors and phagocytosis significantly decreased. Local diabetic foot ulcers are exposed to a high glucose microenvironment for an extended period of time, which reduces the response of macrophages to traumatic stress, causes an early inflammatory response that is relatively slow and prolonged, and an ongoing local inflammatory response that slows the healing process (31). Our findings demonstrated that while pluripotent stem cell abundance was significantly lower in DFUs patients compared to DM patients, macrophage abundance was significantly higher in DFUs patients. The change in the ulcer microenvironment is not due to the time of cell emergence but may be due to the obvious difference in the number and activity of specific cells. This suggests that inflammation and dryness are two important factors affecting DFUs.

Stem cell transplantation has emerged as a novel method of treating diabetic feet in recent years. According to research by Han Y et al., MSCS act in a paracrine manner to promote the development of new blood vessels and granulation tissues by secreting stromal cell-derived factor-1 (SDF-1), vascular epidermal growth factor (VEGF), basic fibroblast growth factor (bFGF), and hepatocyte growth factor (HGF) (32). Encourage the movement of fibroblasts and epithelial keratinocytes to speed up the healing of ulcers. Inhibiting inflammation is another mechanism that aids in diabetic foot healing. The expression of pro-inflammatory factors like TNF-, IL-6, IL-8, IL-1, and ICAM-1 can be inhibited by MSC, while the expression of anti-inflammatory factors like IL-10, high immunomodulatory activity, the regulation of the microenvironment, and the reduction of inflammation can speed up the healing of wounds (33). Our research, however, revealed that in both the healing and non-healing stages of DFUs, there were more hematopoietic stem cells than in DM patients. So, despite the fact that hematopoietic stem cells were numerous in DFUs, we surmised that their activity might be low. In the DFU healing and non-healing stages, both based on the single-cell OCLR algorithm and bulk level, we discovered that the dry score of pluripotent stem cells and hematopoietic stem cells was significantly lower than the dry score of DM. The aforementioned findings suggest that although DFU patients may have some stem cells—possibly even more than healthy individuals—their activity levels are likely to be lower than those of healthy individuals due to the patients’ own conditions, which can make DFUs more difficult to heal or give them a worse prognosis than they would otherwise. For DFU patients, it is essential to find stem cell active agonists as soon as possible.

Further investigation revealed that the main distinct cell populations in the ulcer microenvironment of DM and DFU patients were monocytes, hematopoietic stem cells, and pluripotent stem cells. ANPEP, BID, CYBA, CYBB, FCER1G, ITGA1, and PLAUR were found to be seven drug target genes. To find potential drugs for DFUs treatment, drug-gene and gene-gene drug-gene network maps were created. In one study (34), diabetic rats given the immunosuppressant CYCLOSPORINE showed a proportional decrease in serum glucose levels. In diabetic mice, low-dose preconditioning with SIMVASTATIN boosted angiogenesis, decreased inflammation, and enhanced wound healing (35). An all-natural substance called curcumin has excellent anti-inflammatory and anticancer properties. According to studies, taking nanoccumin significantly improves glycemic control, total cholesterol, low density lipoprotein cholesterol, TAC, and GSH in DFUs patients, but it has no effect on markers of ulcer size (36). Luteolin is a generic term for luteolin. A natural flavonoid called luteolin is present in many plants. Recent research suggests that luteolin significantly lowers blood glucose levels in streptozotocin (STZ)-induced diabetic rats, improves impaired healing, and speeds up skin wound reepithelialization (37). Apigenin’s ability to inhibit glucosidase activity, increase insulin secretion, interact with reactive oxygen species (ROS) in cells, and neutralize them are all factors that together help to prevent diabetic complications. In diabetic and healthy wound tissues, APN GGCH-Hg has a greater effect on wound healing. It exhibits exceptional antioxidant activity. The prepared hydrogel (APN-loaded GGCH-Hg) turns out to have unique biocompatibility, biodegradability, wetting, and antioxidant effects that make it seem to be very suitable for wound healing (38). Chrysin, a naturally occurring flavonoid, has a variety of pharmacological effects, including anti-inflammatory, anti-apoptotic, antioxidant, and anticancer properties. Strong scientific evidence points to albumin’s potential for use in the treatment and prevention of diabetic complications in experimental models (39). Finally, we found that immunofluorescence could identify microenvironment-related markers in skin tissues from six DFUS patients and healthy subjects. The colocalization relationship between CD19, ITGAM, and HLA-DR expression in DFUs skin tissues and monocytes and macrophages of healthy subjects was analyzed by laser confocal microscopy and immunofluorescence triple labeling. The results showed that the expression of CD19, ITGAM, and HLA-DR in the skin of DFUs was significantly higher than in the skin of healthy humans, and that there was a significant relationship between colocalization and expression in DFUs. qPCR was used to find 7 factors whose expression was much higher in DFUs than in healthy people.

At the cellular and molecular levels, this study demonstrates similarities and differences in tissues from various clusters that could serve as potential targets for novel treatments for the advancement of DFUs. Despite the fact that 33 single-cell samples were obtained from data mining, this study was fully validated in 22 DFUs patients and 12 healthy subjects, as well as at the bulk level. Since each cell is a sample, the size of the sample is good enough for the analysis that will come next.

## Conclusions

Significant heterogeneity in the cell clusters was discovered through cluster analysis of cell subsets isolated from normal subjects, diabetic patients, and diabetic foot skin. A higher level of inflammation in DFUs tissues may be predicted by the high expression of macrophages, leukocytes, and monocytes in tissues, whereas a higher level of dryness in diabetes may be predicted by the high proportion of pluripotent stem cells and stromal cells. In addition, stromal cells and pluripotent stem cells showed up later, while other cells were dispersed at all stages in the ulcer microenvironment of diabetes mellitus and DFUs healing and non-healing. Leukocytes, macrophages, and monocytes also showed up earlier. Differences in the number and activity of particular cells are predictive of changes in the DFU microenvironment, rather than the timing of the emergence of particular cells. Between cell ages percentage, DFUs patients had significantly more macrophages than DM patients with pluripotent stem cells did. This suggests even more strongly that dryness and inflammation are key elements influencing DFUs. The results of the overall expression values of hematopoietic or pluripotent stem cells suggest that DFU patients may have a certain number of stem cells, or even more than normal people (possibly related to the body’s self-protection), but that their activity is lower than that of normal people due to the restrictions of their own conditions. DFUs have a poor prognosis in comparison to healthy individuals because it is challenging to heal. ANPEP, BID, CYBA, CYBB, FCER1G, ITGA1, PLAUR, and other genes were significantly overexpressed as drug targets in the DFUs microenvironment. The Drug Gene Network identified CYCLOSPORINE, SIMVASTATIN, CURCUMIN, LUTEOLIN, APIGENIN, and CHRYSIN drugs with potential DFUs.

## Data availability statement

The datasets presented in this study can be found in online repositories. The names of the repository/repositories and accession number(s) can be found in the article/**Supplementary Material**.

## Ethics statement

The studies involving human participants were reviewed and approved by Medical Ethics Committee of Jinshan Hospital affiliated to Fudan University ( JIEC 2021-S44 and IEC-JIEC 2021). The patients/participants provided their written informed consent to participate in this study.

## Author contributions

In this study, all authors participated in the conception and design; YL conceived and wrote the paper; SJ, XL, and WL processed the data; SZ, GW, and YC drew the figures. All authors contributed to the article and approved the submitted version.
